# Routine administration of Anti-D: the ethical case for offering pregnant women fetal *RHD* genotyping and a review of policy and practice

**DOI:** 10.1186/1471-2393-14-87

**Published:** 2014-02-25

**Authors:** Julie Kent, Anne-Maree Farrell, Peter Soothill

**Affiliations:** 1 Department of Health & Social Sciences, University of the West of England, Coldharbour Lane, Bristol BS16 1QY, UK; 2 Faculty of Law, Monash University, Melbourne, Australia; 3 Fetal Medicine, St Michaels Hospital, University Hospitals Bristol NHS Trust, Bristol, UK

**Keywords:** Anti-D immunoglobulin, Fetal RHD genotyping, RhD blood group, Ethics, Informed consent

## Abstract

**Background:**

Since its introduction in the 1960s Anti-D immunoglobulin (Anti-D Ig) has been highly successful in reducing the incidence of haemolytic disease of the fetus and newborn (HDFN) and achieving improvements to maternal and fetal health. It has protected women from other invasive interventions during pregnancy and prevented deaths and damage amongst newborns and is a technology which has been adopted worldwide. Currently about one third of pregnant women with the blood group Rhesus D (RhD) negative in the UK (approximately 40,000 women per year in England and Wales), receive antenatal Anti-D Ig in pregnancy when they do not require it because they are carrying a RhD negative fetus. Since 1997, a test using cell free fetal DNA (cffDNA) in maternal blood has been developed to identify the genotype of the fetus and can be used to predict the fetal RhD blood group.

**Discussion:**

This paper considers whether it is ethically acceptable to continue administering antenatal Anti-D Ig to all RhD negative women when fetal *RHD* genotyping using maternal blood could identify those women who do not need this product.

**Summary:**

The antenatal administration of Anti-D Ig to a third of RhD negative pregnant women who carry a RhD negative fetus and therefore do not need it raises important ethical issues. If fetal *RHD* genotyping using maternal blood was offered to all RhD negative pregnant women it would assist them to make an informed choice about whether or not to have antenatal Anti-D Ig.

## Background

Currently about one third of pregnant women who are blood group RhD negative in the United Kingdom (UK) (approximately 40,000 women per year in England and Wales [1]), receive antenatal Anti-D immunoglobulin (Anti-D Ig) in pregnancy when they do not require it because they are carrying a RhD negative fetus [2]. The purpose of administering Anti-D Ig is to reduce the chance of sensitisation to RhD positive blood and so the potential risks of haemolytic disease of the fetus and newborn (HDFN). Since it is now possible to test maternal blood to predict the RhD blood group of the fetus, our concern is to ask whether it is ethically justifiable for this group of women to continue to be offered Anti-D Ig? Further, while the potential risks of HDFN are well established, and the risks of receiving Anti-D Ig are very low, should we accept that pregnant women and their RhD negative fetuses are exposed to a plasma product sourced from non-local, overseas donors where there are no clinical or other benefits to the woman or the fetus?

## Discussion

### Administration of Anti-D Ig

Since its introduction in the 1960s Anti-D Ig has been highly successful in reducing the incidence of HDFN and achieving improvements to maternal and fetal health. It has protected women from other invasive interventions during pregnancy and prevented deaths and damage amongst newborns and is a technology which has been adopted worldwide. Yet there are international and local variations in the pattern of its administration [3]. In accordance with current UK NICE guidelines, [4] it is recommended that all RhD negative women in the UK are routinely offered Anti-D Ig prophylactically (ie routine antenatal anti-D prophylaxis - RAADP) during antenatal care (at 28 weeks), and following any antenatal ‘potential sensitising event’ (PSE) where fetal maternal haemorrhage (FMH) may have occurred (including pregnancy termination). Anti-D Ig is also offered after the delivery but only when a cord blood test shows that the baby is RhD positive. The above policies are designed to prevent sensitisation in RhD negative women who carry a RhD positive fetus and so antibody production arising to HDFN in subsequent pregnancies with RhD positive fetuses. The success of the above programme has led to the incidence of HDFN now being relatively uncommon, although some concern has been expressed about recent cases [5]. If the fetus is RhD negative then in that pregnancy sensitisation and associated HDFN due to anti-D cannot occur [6].

### Non invasive prenatal testing of fetal genotype

Since 1997, [7] a test using cell free fetal DNA (cffDNA) in maternal blood has been developed to identify the genotype of the fetus and can be used to predict the fetal RhD blood group. This was initially used in women who had already been sensitised and so were at risk of HDFN [8,9], however, from 2008 modifications were made to allow a scaled-up high throughput approach [10]. Standardisation of the test has been the focus of international research, and it has been shown to be very accurate but the small possibility of false negative results remains. If the test gave a false negative result and routine cord blood phenotype testing at birth subsequently identified the fetus as RhD positive then postnatal Anti-D Ig would still be administered at that time, but potentially sensitisation could occur in these women affecting subsequent pregnancies. The risks of this happening have been estimated to be 1:86,000 [2]. In the UK, non-invasive (or invasive) fetal blood group genotyping is currently only performed when women’s samples are referred to the NHS Blood and Transplant (NHSBT) or Scottish National Blood Transfusion Service (SNBTS) [11] for testing to determine the risk to the fetus when a mother is known to have immune antibodies against the relevant blood group antigen. A multicentre “research for patient benefit” NIHR project has finished recruiting and is awaiting publication. In addition a service implementation pilot that offers a fetal genotyping test to all RhD negative women is now underway in Bristol and Weston and results are expected in late 2014 [12]. Only the Netherlands and Denmark currently offer this test to all RhD negative women in order to identify those carrying a fetus that is RhD negative and to reduce the unnecessary administration of Anti-D Ig [13,14]. In Denmark, the decision was made concurrently with the implementation of RAADP and it was recognised that the accuracy of fetal *RHD* genotyping was similar to that of the cord blood RhD phenotyping used for the administration of postnatal Anti-D Ig. In the Netherlands, RAADP had already been established when fetal *RHD* genotyping began to be offered to all RhD negative women in 2011.

### The production of Anti-D Ig

In the UK, polyclonal Anti-D Ig is a blood product manufactured from pooled plasma, predominantly collected from RhD negative male plasma donors in the United States. These male donors are injected with RhD positive red blood cells to stimulate sensitisation and antibody production. The antibodies can then be harvested following plasmapheresis. A premium is paid to these men in acknowledgement of the potential risks they face as a result of injecting donor red blood cells prior to the donation session. The processing and fractionation of plasma operates to industry standards and must comply with medicinal product regulation in order to minimise the risks of infection or viral transmission or contamination [15].

In the 1970s and 1990s, there were a number of contamination episodes involving Anti-D Ig product in countries such as Ireland and Germany [16,17]. More recently, the UK Royal College of Obstetricians and Gynaecologists noted that: “There is no evidence to suggest that RAADP is associated with adverse events that are of consequence for the mother or baby, other than *the possibility of blood-borne infection* (author’s emphasis), and procedures are in place to minimise these risks” and to inactivate viruses [18]. What is particularly troubling is that the risks of prion transmission and newly emerging viruses are unknown and therefore remain a potential risk for women who continue to receive the product. In addition, administration of Anti-D Ig and adverse incidents relating to its use are a matter of concern for organisations such as Serious Hazards of Transfusion, [19] and concerns have recently been raised about adverse incidents involving the inappropriate and unnecessary administration of Anti-D Ig to women who are RhD positive.

### Ethical issues

Anti-D therapy has no direct benefit to the woman but is designed to promote fetal health in future pregnancies. The ethical (and legal) basis for current policy and practice is that women should be given appropriate information about Anti-D Ig so that they are in a position to give consent to the treatment. But on what basis are women able to make such a decision when the RhD blood group of their fetus remains unknown? Introduction of fetal *RHD* genotyping to prevent unnecessary administration of Anti-D Ig would be more consistent with existing policy which is aimed at reducing wasteful use of blood and blood products and ensuring that the right product is given to the right person [20]. Moreover, while a recently published cost analysis of mass fetal *RHD* genotyping suggested that the costs of introducing such a service would not be met by the reduction in use of prophylactic antenatal Anti-D Ig, [21] others have argue that automated testing lowers assay costs below the price of Anti-D Ig and is cost-effective [22]. There are inconsistencies in the application of cost-analysis decision making within the NHS. The highly precautionary approach now taken to managing risks in a post-HIV blood contamination era has meant that a range of values – ethical, social and political – have influenced policy-making in relation to blood safety, rather than relying predominantly on traditional cost-benefit analysis in relation to healthcare interventions [23]. So what values should underpin the policy that about 40,000 women per year in England and Wales continue to receive a blood product they do not need?

We want to suggest that in addition to the audit of Anti-D Ig use in England that is being undertaken by the Royal College of Physicians and NHSBT as part of their joint National Comparative Audit (NCA) program, a review of the ethical issues involved is also needed [24]. Does the well accepted ethical principle that clinicians should do no harm encompass the view that women should not be given a blood product that does them no good? Does consent to the administration of Anti-D Ig also mean that women should first be offered fetal *RHD* genotyping to establish their need for this product? Current policy and practice in the UK to routinely administer Anti-D Ig emerged at a time when the technology of fetal *RHD* genotyping was under-developed but a review of the current approach is now timely given the development of new capabilities to test fetal genotype. The widespread adoption of the technology could ensure that women, both in the UK and in other countries, do not receive a blood product unnecessarily. The ethical issues at stake here include weighing the relatively low risks of false negative results associated with fetal *RHD* genotyping using cffDNA and the costs of implementing mass testing, against the benefits of ceasing the practice of giving this group of pregnant women a human blood product they do not need.

## Summary

In short we have argued that, on ethical grounds, there is a strong case for reviewing policy and practice relating to the routine administration of prophylactic antenatal Anti-D Ig. By making fetal RHD genotyping more widely available, women would be better informed about whether or not they need this blood product.

## Competing interests

The authors declare that they have no competing interests.

## Authors’ contributions

JK contributed to the design of the ESRC study, conducted fieldwork, including interviews, analysed the data and reviewed the literature, developed the idea for the paper and wrote the paper. AMF contributed to the design of the ESRC study, the analysis of the data and literature, commenting on the paper and assisting in its revision. PS provided information about current clinical practice, commented on the paper and assisted in its revision. All authors approved the final version of the paper.

## Authors’ information

JK is Professor of Sociology of Health Technology and is researching the plasma industry and the use of blood products in maternity care as part of an ESRC funded project: *Risk, safety and consent in blood services in the UK*. She is author of *Regenerating Bodies: Tissue and Cell Therapies in the 21*^*st*^*Century* (London: Routledge; 2012). AMF is Associate Professor of Law, Australian Research Council Future Fellow, and collaborator with JK on the ESRC project (above). She is author of *The Politics of Blood, Ethics, Innovation and the Regulation of Risk* (Cambridge: Cambridge University Press; 2012). PS is a Consultant and Professor of Maternal and Fetal Medicine and a lead for the service implementation pilot of fetal *RHD* genotyping at Bristol and Weston. He was a grant holder of the NIHR funded project on Reliable Accurate Prenatal non-Invasive Diagnosis (RAPID) http://www.rapid.nhs.uk and National Institute for Health Research, Research for Patient Benefit Programme Grant (PB-PG-0107-12005).

## Pre-publication history

The pre-publication history for this paper can be accessed here:

http://www.biomedcentral.com/1471-2393/14/87/prepub
